# Adherence to Treatment in Allergic Rhinitis During the Pollen Season in Europe: A MASK‐air Study

**DOI:** 10.1111/cea.70004

**Published:** 2025-02-16

**Authors:** Bernardo Sousa‐Pinto, Elísio M. Costa, Rafael José Vieira, Ludger Klimek, Wienczyslawa Czarlewski, Oliver Pfaar, Anna Bedbrook, Rita Amaral, Luisa Brussino, Violeta Kvedariene, Desiree E. Larenas‐Linnemann, Tomohisa Iinuma, Nhân Pham‐Thi, Frederico S. Regateiro, Luis Taborda‐Barata, Maria Teresa Ventura, Ignacio J. Ansotegui, Karl C. Bergmann, G. Walter Canonica, Victoria Cardona, Lorenzo Cecchi, Ivan Cherrez‐Ojeda, Cemal Cingi, Alvaro A. Cruz, Stefano Del Giacco, Philippe Devillier, Wytske J. Fokkens, Bilun Gemicioglu, Tari Haahtela, Juan Carlos Ivancevich, Piotr Kuna, Helga Kraxner, Daniel Laune, Renaud Louis, Michael Makris, Mario Morais‐Almeida, Ralph Mösges, Marek Niedoszytko, Nikolaos G. Papadopoulos, Vincenzo Patella, Ana Margarida Pereira, Sietze Reitsma, Karla Robles‐Velasco, Philip W. Rouadi, Boleslaw Samolinski, Milan Sova, Sanna K. Toppila‐Salmi, Joaquin Sastre, Arunas Valiulis, Arzu Yorgancioglu, Mihaela Zidarn, Torsten Zuberbier, Joao A. Fonseca, Jean Bousquet, the MASK‐air think tank, Josep M. Anto, Maciej Kupczyk, Marek Kulus, Nicolas Roche, Nicola Scichilone, Rute Almeida, Sinthia Bosnic‐Anticevich, Fulvio Braido, Claudia Chaves Loureiro, Govert de Vries, Antonio F. M. Giuliano, Cristina Jácome, Igor Kaidashev, Gilles Louis, Olga Lourenço, Mika Makela, Marcus Maurer, Joaquim Mullol, Rachel Nadif, Robyn O’Hehir, Yoshitaka Okamoto, Markus Ollert, Heidi Olze, Benoit Pétré, Francesca Puggioni, Jan Romantowski, Daniela Rivero‐Yeverino, Monica Rodriguez‐Gonzalez, Ana Sá‐Sousa, Marine Savouré, Faradiba S. Serpa, Mohamed H. Shamji, Aziz Sheikh, Charlotte Suppli Ulrik, Mikhail Sofiev, Annette Sperl, Ana Todo‐Bom, Ioanna Tsiligianni, Erkka Valovirta, Michiel van Eerd, Hubert Blain, Louis‐Philippe Boulet, Guy Brusselle, Roland Buhl, Denis Charpin, Thomas Casale, Tomas Chivato, Jaime Correia‐de‐Sousa, Christopher Corrigan, Frédéric de Blay, Mark Dykewicz, Alessandro Fiocchi, Mattia Giovannini, Ewa Jassem, Marek Jutel, Thomas Keil, Stefania La Grutta, Brian Lipworth, Alberto Papi, Jean‐Louis Pépin, Santiago Quirce, Carlos Robalo Cordeiro, Maria J. Torres, Omar S. Usmani, Matteo Bonini, Brigita Gradauskiene, Christopher Brightling

**Affiliations:** ^1^ MEDCIDS–Department of Community Medicine, Information and Health Decision Sciences, Faculty of Medicine University of Porto Porto Portugal; ^2^ CINTESIS@RISE–Centre for Health Technology and Services Research, Health Research Network, Faculty of Medicine University of Porto Porto Portugal; ^3^ CINTESIS@RISE, Biochemistry Lab, Faculty of Pharmacy and Competence Center on Active and Healthy Ageing University of Porto Porto Portugal; ^4^ Department of Otolaryngology, Head and Neck Surgery Universitätsmedizin Mainz Mainz Germany; ^5^ Center for Rhinology and Allergology Wiesbaden Germany; ^6^ Medical Consulting Czarlewski Levallois France; ^7^ ARIA Montpellier France; ^8^ Section of Rhinology and Allergy, Department of Otorhinolaryngology, Head and Neck Surgery, University Hospital Marburg Philipps‐Universität Marburg Marburg Germany; ^9^ MASK‐Air SAS Montpellier France; ^10^ Department of Women's and Children's Health, Paediatric Research Uppsala University Uppsala Sweden; ^11^ Department of Cardiovascular and Respiratory Sciences, Porto Health School Polytechnic Institute of Porto Porto Portugal; ^12^ Department of Medical Sciences University of Torino Torino Italy; ^13^ Allergy and Clinical Immunology Unit Mauriziano Hospital Torino Italy; ^14^ Institute of Clinical Medicine, Clinic of Chest Diseases and Allergology, Faculty of Medicine Vilnius University Vilnius Lithuania; ^15^ Institute of Biomedical Sciences, Department of Pathology, Faculty of Medicine Vilnius University Vilnius Lithuania; ^16^ Center of Excellence in Asthma and Allergy Médica Sur Clinical Foundation and Hospital México City Mexico; ^17^ Department of Otorhinolaryngology Chiba University Hospital Chiba Japan; ^18^ Ecole Polytechnique de Palaiseau Palaiseau France; ^19^ IRBA (Institut de Recherche Bio‐Médicale des Armées) Bretigny sur Orge France; ^20^ Université Paris Cité Paris France; ^21^ Allergy and Clinical Immunology Department, Hospitais da Universidade de Coimbra Unidade Local de Saúde de Coimbra Coimbra Portugal; ^22^ Center for Innovative Biomedicine and Biotechnology (CIBB), Faculty of Medicine University of Coimbra Coimbra Portugal; ^23^ Institute of Immunology, Faculty of Medicine University of Coimbra Coimbra Portugal; ^24^ UBIAir–Clinical & Experimental Lung Centre and CICS‐UBI Health Sciences Research Centre University of Beira Interior Covilhã Portugal; ^25^ Department of Immunoallergology Cova da Beira University Hospital Centre Covilhã Portugal; ^26^ University of Bari Medical School Bari Italy; ^27^ Institute of Sciences of Food Production National Research Council (ISPA‐CNR) Bari Italy; ^28^ Department of Allergy and Immunology Hospital Quironsalud Bizkaia Bilbao Spain; ^29^ Institute of Allergology, Charité–Universitätsmedizin Berlin Corporate Member of Freie Universität Berlin and Humboldt‐Universität zu Berlin Berlin Germany; ^30^ Fraunhofer Institute for Translational Medicine and Pharmacology ITMP, Immunology and Allergology Berlin Germany; ^31^ Department of Biomedical Sciences Humanitas University, Pieve Emanuele Milan Italy; ^32^ Asthma and Allergy Unit IRCCS Humanitas Research Hospital, Rozzano Milan Italy; ^33^ Allergy Section, Department of Internal Medicine Hospital Vall d'Hebron Barcelona Spain; ^34^ ARADyAL Research Network Barcelona Spain; ^35^ SOS Allergology and Clinical Immunology USL Toscana Centro Prato Italy; ^36^ Universidad Espíritu Santo Samborondón Ecuador; ^37^ Respiralab Research Group Guayaquil Guayas Ecuador; ^38^ Medical Faculty, ENT Department Eskisehir Osmangazi University Eskisehir Turkey; ^39^ Fundaçao ProAR Federal University of Bahia and GARD/WHO Planning Group Salvador Bahia Brazil; ^40^ Department of Medical Sciences and Public Health and Unit of Allergy and Clinical Immunology, University Hospital “Duilio Casula” University of Cagliari Cagliari Italy; ^41^ VIM Suresnes, UMR 0892, Pôle des Maladies des Voies Respiratoires, Hôpital Foch Université Paris‐Saclay Suresnes France; ^42^ Department of Otorhinolaryngology Amsterdam University Medical Centres, AMC Amsterdam the Netherlands; ^43^ Department of Pulmonary Diseases, Istanbul University‐Cerrahpaşa Cerrahpaşa Faculty of Medicine Istanbul Turkey; ^44^ Institute of Pulmonology and Tuberculosis Istanbul University‐Cerrahpaşa Istanbul Turkey; ^45^ Skin and Allergy Hospital Helsinki University Hospital, and University of Helsinki Helsinki Finland; ^46^ Servicio de Alergia e Immunologia Clinica Santa Isabel Buenos Aires Argentina; ^47^ Division of Internal Medicine, Asthma and Allergy, Barlicki University Hospital Medical University of Lodz Lodz Poland; ^48^ Department of Otorhinolaryngology, Head and Neck Surgery Semmelweis University Budapest Hungary; ^49^ KYomed INNOV Montpellier France; ^50^ Department of Pulmonary Medicine CHU Liège Liège Belgium; ^51^ GIGA I3 Research Group University of Liège Liège Belgium; ^52^ Allergy Unit “D Kalogeromitros”, 2nd Dpt of Dermatology and Venereology, National & Kapodistrian University of Athens “Attikon” University Hospital Athens Greece; ^53^ Allergy Center CUF Descobertas Hospital Lisbon Portugal; ^54^ Institute of Medical Statistics and Computational Biology University of Cologne Cologne Germany; ^55^ ClinCompetence Cologne GmbH Cologne Germany; ^56^ Department of Allergology Medical University of Gdańsk Gdansk Poland; ^57^ Allergy Department, 2nd Pediatric Clinic University of Athens Athens Greece; ^58^ Division of Allergy and Clinical Immunology, Department of Medicine 'Santa Maria della Speranza' Hospital, Battipaglia Salerno Italy; ^59^ Agency of Health ASL Salerno Italy; ^60^ Postgraduate Program in Allergy and Clinical Immunology University of Naples Federico II Naples Italy; ^61^ PaCeIT–Patient Centered Innovation and Technologies, Center for Health Technology and Services Research (CINTESIS), Faculty of Medicine University of Porto Porto Portugal; ^62^ Department of Otolaryngology, Head and Neck Surgery Eye and Ear University Hospital Beirut Lebanon; ^63^ Department of Otorhinolaryngology, Head and Neck Surgery Dar Al Shifa Hospital Salmiya Kuwait; ^64^ Department of Prevention of Environmental Hazards, Allergology and Immunology Medical University of Warsaw Warsaw Poland; ^65^ Department of Respiratory Medicine and Tuberculosis University Hospital Brno Czech Republic; ^66^ Department of Otorhinolaryngology University of Eastern Finland and the North Savo Wellbeing Services County Kuopio Finland; ^67^ Department of Allergy, Skin and Allergy Hospital, Inflammation Center Helsinki University Hospital and University of Helsinki Helsinki Finland; ^68^ Allergy Service, Fundacion Jimenez Diaz Universidad Autonoma de Madrid, CIBERES‐ISCIII Madrid Spain; ^69^ Interdisciplinary Research Group of Human Ecology, Institute of Clinical Medicine and Institute of Health Sciences Medical Faculty of Vilnius University Vilnius Lithuania; ^70^ Clinic of Children's Diseases, Institute of Clinical Medicine and Institute of Health Sciences Medical Faculty of Vilnius University Vilnius Lithuania; ^71^ Clinic of Asthma, Allergy, and Chronic Lung Diseases Vilnius Lithuania; ^72^ Department of Pulmonary Diseases Celal Bayar University, Faculty of Medicine Manisa Turkey; ^73^ Faculty of Medicine University of Ljubljana Ljubljana Slovenia; ^74^ University Clinic of Respiratory and Allergic Diseases Golnik Slovenia

**Keywords:** allergic rhinitis, mobile health, treatment adherence

## Abstract

**Background:**

Adherence to rhinitis treatment has been insufficiently assessed. We aimed to use data from the MASK‐air mHealth app to assess adherence to oral antihistamines (OAH), intra‐nasal corticosteroids (INCS) or azelastine‐fluticasone in patients with allergic rhinitis.

**Methods:**

We included regular European MASK‐air users with self‐reported allergic rhinitis and reporting at least 1 day of OAH, INCS or azelastine‐fluticasone. We assessed weeks during which patients answered the MASK‐air questionnaire on all days. We restricted our analyses to data provided between January and June, to encompass the pollen seasons across the different assessed countries. We analysed symptoms using visual analogue scales (VASs) and the combined symptom‐medication score (CSMS), performing stratified analyses by weekly adherence levels. Medication adherence was computed as the proportion of days in which patients reported rhinitis medication use. Sensitivity analyses were performed considering all weeks with at most 1 day of missing data and all months with at most 4 days of missing data.

**Results:**

We assessed 8212 complete weeks (1361 users). Adherence (use of medication > 80% days) to specific drug classes ranged from 31.7% weeks for azelastine‐fluticasone to 38.5% weeks for OAH. Similar adherence to rhinitis medication was found in users with or without self‐reported asthma, except for INCS (better adherence in asthma patients). VAS and CSMS levels increased from no adherence to full adherence, except for INCS. A higher proportion of days with uncontrolled symptoms was observed in weeks with higher adherence. In full adherence weeks, 41.2% days reported rhinitis co‐medication. The sensitivity analyses displayed similar results.

**Conclusions:**

A high adherence was found in patients reporting regular use of MASK‐air. Different adherence patterns were found for INCS compared to OAH or azelastine‐fluticasone that are likely to impact guidelines.

## Graphical abstract

In a study assessing mobile health data, (i) adherence to rhinitis medication was highest for oral antihistamines and lowest for azelastine‐fluticasone, (ii) in weeks of partial adherence, azelastine‐fluticasone was associated with lower levels of reported rhinitis symptoms than other medication classes.

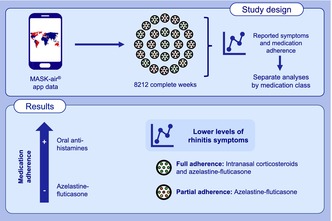

**Summary**

- In patients regularly using the MASK‐air app, a high adherence was found for all medications.
- Different adherence patterns were found for intranasal corticosteroids compared to oral antihistamines or intranasal azelastine‐fluticasone.
- In weeks in which patients display poorer rhinitis control, adherence to medication tends to increase.

## 1 Introduction

The current strategy for allergic rhinitis management is centred around continuous long‐term treatments, whose effectiveness has usually been assessed based on randomised controlled trials (RCTs) [1, 2]. However, in most of these RCTs, adherence is high and does not reflect real‐life situations. In fact, both the physicians' experience and available real‐world data strongly suggest that patients are not very adherent to rhinitis medications, prompting the need for a quantitative assessment of adherence in real‐world settings [3]. One approach for assessing adherence involves the use of electronic devices that count and record the drugs taken. However, these devices are expensive and, as such, not a viable solution for large studies in rhinitis patients and for daily clinical practice [4].

Although guidelines are largely based on the ‘high‐adherence RCT‐like scenarios’, such scenarios are not common in everyday clinical practice [5, 6]. Understanding adherence patterns to rhinitis medication may enable the proposition of treatment strategies tailored to such patterns (e.g., proposing an as‐needed treatment depending on symptoms, rather than the classical continuous treatment) [7].

Some mHealth studies have also been carried out to assess adherence. These include studies using MASK‐air, a mobile health app assessing the daily control of allergic rhinitis and asthma, which is freely available and has currently been launched in 29 countries [8]. It has been classified as a Good Practice of the Directorate General Health and Food Safety (European Commission) for digitally enabled, patient‐centred care in rhinitis and asthma multimorbidity [9]. It is also one of the 13 Organisation of Economic Cooperation and Development (OECD) Best Practices in integrated care for chronic diseases [10]. The MASK‐air mHealth app has been used to assess adherence to medications in a group of users reporting from 7 to 15 days of app use at any time (i.e., not necessarily consecutive days). In that study, more than 75% of patients were non‐adherent to medications [11]. However, in this study, consecutive periods of MASK‐air reporting were not necessarily reported and the overrepresentation of isolated days may therefore occur on which patients displayed particularly severe symptoms and were more eager to use the MASK‐air app.

In this study, we used longitudinal periods (weeks or months) of MASK‐air mHealth real‐life data to assess adherence to overall rhinitis medication and to three major classes of rhinitis medications (oral anti‐histamines [OAH], intranasal corticosteroids [INCS] and the fixed intranasal combination of azelastine and fluticasone propionate [AzeFlu]). In addition, we also assessed the association between medication adherence and reported rhinitis control.

## 2 Methods

### 2.1 Study Design

Using the MASK‐air database, we analysed data from European users with self‐reported allergic rhinitis and ever use of any rhinitis medication, OAH, INCS or AzeFlu (as this was the only fixed combination available when the study started). We analysed all weeks during which patients answered to the MASK‐air daily monitoring questionnaire on all days, assessing medication adherence to any rhinitis medication, OAH, INCS and AzeFlu. In addition, we analysed reported symptoms, performing stratified analyses by weekly adherence levels. A sensitivity analysis was performed considering (i) all weeks during which patients had at most one missing day of MASK‐air reporting and (ii) all months with at most 4 days of missing data.

### 2.2 Setting and Participants

The MASK‐air app [8] was launched in 2015 and is freely available on the Google Play and Apple App Stores. It is available in 29 countries [8]. We included data (May 21, 2015—December 31, 2022) from European MASK‐air users older than the age of digital consent (which ranges between 13 and 16 years depending on the country), with self‐reported allergic rhinitis and who reported at least 1 day of any rhinitis medication, OAH, INCS or AzeFlu use. In our main analyses, we assessed all weeks (sets of seven consecutive days) during which patients answered to the MASK‐air daily monitoring questionnaire on all days. We considered data provided from January 1 to June 30, in order to encompass the pollen seasons across the different assessed countries, according to previous studies. We performed sensitivity analyses considering (i) all weeks during which patients had at most one missing day of MASK‐air reporting and (ii) all months during which patients had at most four missing days of MASK‐air reporting.

### 2.3 Ethics

MASK‐air complies with the General Data Protection Regulation. The use of MASK‐air data for research purposes has been approved by an independent review board (Köln‐Bonn, Germany). Users consented to having their data analysed for scientific purposes in the terms of use of the app. All data were provided by users anonymously.

### 2.4 Data Sources and Variables

MASK‐air includes a daily monitoring questionnaire assessing (i) the impact of allergy symptoms through four mandatory visual analogue scales (VASs) on a 0–100 scale, in which higher values indicate a worse impact (Table S1) and (ii) the rhinitis daily medication use (available from country‐specific lists with prescribed and over‐the‐counter medications).

Symptom and medication data daily provided by patients allow the calculation of the allergy combined symptom‐medication score (CSMS), according to the following previously published formula: [12].

$$\begin{array}{c} \begin{array}{c} {}[\left(0.037\times \mathrm{VAS}\;\text{Global Symptoms}\right)+\left(0.033\times \mathrm{VAS}\;\text{Eyes}\right)\\ \;+\left(0.020\times \mathrm{VAS}\;\text{Nose}\right)+\left(0.027\times \mathrm{VAS}\;\text{Asthma}\right)\\ \;+\left(0.450\;\text{if AzeFlu is used}\right)+\left(0.424\;if\;\text{nasal steroids}\;\mathrm{are}\;\text{used}\right)\\ \;+\left(0.243\;\text{if asthma medication is used}\right)\\ \;+\left(0.380\;if\;\text{other rhinitis relief medication is used}\right)]\times 7.577 \end{array} \end{array}$$

The responses to the daily questionnaire allow the computation of medication adherence. In particular, when considering all rhinitis medications, adherence was calculated as the proportion of reported MASK‐air days on which any rhinitis medication was used. For specific drug classes, adherence was calculated as the proportion of reported MASK‐air days on which a medication of that class was used. We considered that there was medication adherence for the weeks in which the self‐reported use of rhinitis medication occurred in > 80% of days [13].

### 2.5 Sample Size

We analysed all complete weeks from users meeting the eligibility criteria. No sample size calculation was performed.

### 2.6 Statistical Analysis

When responding to the MASK‐air daily monitoring questionnaire, it is not possible to skip any of the questions, and data are saved to the dataset only after the final answer. This precludes any missing data within each questionnaire.

Categorical variables were described using absolute and relative frequencies, while continuous variables were described using medians and interquartile ranges (IQRs). For comparison between different age groups, effect size measures for differences in proportions and medians were estimated. Effect size measures < 0.2 indicate non‐meaningful differences, between 0.2 and 0.5 small differences, between 0.5 and 0.8 moderate differences and higher than 0.8 large differences [14].

For overall rhinitis medications and for each drug class, we assessed the frequency of weeks in which there was no adherence (i.e., drugs not being used on any of the days), partial adherence or adherence (i.e., drugs being used on > 80% of the days). In addition, we assessed the median and maximum VAS and CSMS levels in weeks with no adherence, partial adherence and full adherence. An additional analysis was performed for individual INCS considering medications reported in at least 1000 weeks of data in MASK‐air.

For overall rhinitis medications and for each drug class, we also assessed the frequency of not well controlled days (VAS nose > 20 or VAS eye > 20) and the frequency of co‐medication days in weeks with no adherence, partial adherence and full adherence. To assess how symptoms may influence medication adherence, we built multivariable mixed‐effects linear regression models with (i) the dependent variable corresponding to the percentage of weekly days using rhinitis medication, OAH, INCS or AzeFlu and (ii) independent variables corresponding to the number of not well controlled days regarding nasal or ocular symptoms. Observations were clustered by patient (i.e., the patient was set as a random‐effect).

We performed sensitivity analyses considering (i) weeks with at most one missing day of MASK‐air reporting and (ii) months with at most 4 days of missing data.

All analyses were performed using software R.

## 3 Results

### 3.1 Sample Characteristics

We assessed a total of 8212 complete weeks provided by 1361 MASK‐air users reporting at least 1 day of rhinitis medication use. This comprised 1216 users reporting at least 1 day of OAH use (7405 weeks), 630 users reporting at least 1 day of INCS use (4090 weeks) and 316 users reporting at least 1 day of AzeFlu use (2334 weeks) (Table 1; Tables S2 and S3; Figure S1). The mean participants' age was 40.5 years (standard‐deviation = 13.8). 4099 weeks (49.9%) were provided by women. For sensitivity analyses, we assessed 11,389 weeks (1794 users) with at most 1 day of missing MASK‐air reporting and 1283 months (484 users) with at most 4 days of missing data.

**TABLE 1:** Demographic and clinical characteristics of the assessed MASK‐air users.

	All rhinitis medications^a^	Oral antihistamines	Intranasal corticosteroids	Azelastine‐fluticasone
Complete weeks of MASK‐air reporting				
*N* weeks [*N* users]	8212 [1361]	7405 [1216]	4090 [630]	2334 [316]
Females–*N* (%)	4099 (49.9)	3747 (50.6)	2207 (54.0)	1257 (53.9)
Age–mean (SD)	40.5 (13.8)	40.2 (13.5)	41.9 (14.0)	40.0 (13.6)
VAS nose–median (IQR)	11 (20)	11 (20)	12 (20)	12 (19)
VAS eye–median (IQR)	4 (14)	4 (14)	5 (15)	3 (12)
CSMS–median (IQR)	9.0 (14.4)	8.9 (15.0)	10.4 (14.3)	10.9 (14.2)
Self‐reported asthma–*N* (%)	3290 (40.1)	2925 (39.5)	1767 (43.2)	1120 (48.0)
Conjunctivitis–*N* (%)	6183 (75.3)	5701 (77.0)	3242 (79.3)	1684 (72.2)
Weeks with 6 or 7 days of MASK‐air reporting				
*N* weeks [*N* users]	11,389 [1794]	10,193 [1572]	5623 [824]	3156 [420]
Females–*N* (%)	5669 (49.8)	5163 (50.7)	2978 (53.0)	1703 (54.0)
Age–mean (SD)	40.3 (13.9)	40.0 (13.7)	41.3 (14.0)	39.3 (13.7)
VAS nose–median (IQR)	11 (21)	11 (21)	13 (21)	12 (20)
VAS eye–median (IQR)	4 (15)	5 (15)	5 (16)	4 (13)
CSMS–median (IQR)	9.3 (15.1)	9.2 (15.6)	10.7 (14.8)	11.1 (14.3)
Self‐reported asthma–*N* (%)	4608 (40.5)	4066 (39.9)	2493 (44.3)	1483 (47.0)
Conjunctivitis–*N* (%)	8550 (75.1)	7843 (76.9)	4407 (78.4)	2286 (72.4)
Months with at most 4 missing days of MASK‐air reporting				
*N* months [*N* users]	1283 [484]	1161 [437]	657 [216]	383 [113]
Females–*N* (%)	612 (47.7)	559 (48.1)	349 (53.1)	197 (51.4)
Age–mean (SD)	41.1 (13.7)	40.6 (13.2)	42.9 (14.1)	41.7 (13.5)
VAS nose–median (IQR)	10 (18)	10 (19)	12 (19)	12 (20)
VAS eye–median (IQR)	3 (12)	3 (12)	4 (14)	3 (12)
CSMS–median (IQR)	8.1 (13.1)	7.9 (13.8)	9.8 (13.4)	11.1 (14.4)
Self‐reported asthma–*N* (%)	511 (39.8)	456 (39.3)	281 (42.8)	177 (46.2)
Conjunctivitis–*N* (%)	981 (76.5)	908 (78.2)	533 (81.1)	280 (73.1)

Abbreviations: CSMS = combined symptom‐medication score, IQR = interquartile range, SD = standard deviation, VAS = visual analogue scale.

^a^ Group corresponding to patients using any kind of rhinitis medication and, therefore, not corresponding to the sum of weeks and users using oral antihistamines, intranasal corticosteroids and azelastine‐fluticasone.

### 3.2 Adherence to Rhinitis Medication

In the main analysis considering complete weeks, we observed that there was adherence to any rhinitis medication (i.e., use of any rhinitis medication > 80% days) in 50.1% of the weeks. When considering drug classes, adherence was observed in a lower frequency of weeks, ranging from 31.7% (AzeFlu) to 38.5% (OAH) (Table 2; Figure S2). Similar results were observed when considering weeks with 6 or 7 days of reporting or monthly data (Tables S4 and S5).

**TABLE 2:** Adherence in patients who reported ever use of rhinitis medication, oral antihistamines (OAH), intranasal corticosteroids (INCS) and azelastine‐fluticasone (AzeFlu) in complete weeks (weeks with all days of MASK‐air reporting).

	All rhinitis medications^a^	OAH	INCS	AzeFlu	Effect size		
					OAH vs. INCS	OAH vs. AzeFlu	INCS vs. AzeFlu
Adherence classes–*N* weeks (%)							
0%	2362 (28.8)	2992 (40.4)	1862 (45.5)	1235 (52.9)	0.10	0.25	0.15
1%–40%	838 (10.2)	816 (11.0)	356 (8.7)	176 (7.5)	0.08	0.12	0.04
41%–80%	895 (10.9)	748 (10.1)	398 (9.7)	182 (7.8)	0.01	0.08	0.07
> 80%	4117 (50.1)	2849 (38.5)	1474 (36.0)	741 (31.7)	0.05	0.14	0.09
Weekly median VAS nose per adherence class–median (IQR)							
0%	4 (13)	6 (15)	10 (20)	11 (19)	0.33	0.41	0.07
1%–40%	9 (18)	11 (19)	14 (18)	11 (16)	0.19	0	0.21
41%–80%	11 (17)	13 (21)	16 (22)	14 (20)	0.21	0.04	0.18
> 80%	15 (24)	16 (25)	14 (19)	14 (25)	0.13	0.14	0
Weekly maximum VAS nose per adherence class—median (IQR)							
0%	11 (24)	14 (25)	19 (32)	20 (27)	0.27	0.33	0.05
1%–40%	26 (32)	28 (34)	28 (35)	25 (28)	0	0.14	0.14
41%–80%	26 (36)	29 (38)	31 (36)	25 (38)	0.08	0.16	0.25
> 80%	27 (37)	30 (37)	23 (32)	27 (37)	0.29	0.20	0.09
Weekly median VAS eye per adherence class–median (IQR)							
0%	0 (7)	0 (8)	3 (13)	3 (10)	0.95	0.95	0
1%–40%	3 (10)	4 (12)	6 (13)	4 (14)	0.26	0	0.26
41%–80%	5 (13)	7 (19)	7 (18)	4 (14)	0.05	0.31	0.35
> 80%	7 (20)	8 (20)	7 (19)	5 (15)	0.09	0.30	0.22
Weekly maximum VAS eye per adherence class–median (IQR)							
0%	5 (17)	6 (19)	9 (24)	7 (21)	0.27	0.10	0.17
1%–40%	13 (26)	15 (27)	15 (28)	9 (26)	0	0.38	0.40
41%–80%	13 (30)	18 (36)	16 (38)	10 (31)	0.12	0.44	0.31
> 80%	16 (34)	19 (37)	15 (30)	14 (31)	0.20	0.24	0.05
Weekly median CSMS per adherence class–median (IQR)							
0%	3.4 (9.1)	5.1 (11.1)	7.0 (13.6)	8.8 (13.6)	0.24	0.41	0.18
1%–40%	6.6 (11.1)	8.0 (12.0)	9.2 (12.4)	9.9 (13.9)	0.14	0.21	0.08
41%–80%	9.3 (12.7)	10.5 (14.9)	12.7 (15.5)	12.5 (14.2)	0.22	0.39	0.18
> 80%	12.8 (16.2)	13.4 (17.4)	13.2 (14.0)	14.7 (17.0)	0.02	0.08	0.07
Weekly maximum CSMS per adherence class–median (IQR)							
0%	7.1 (14.0)	9.2 (15.7)	12.2 (19.2)	13.7 (16.7)	0.25	0.39	0.12
1%–40%	16.4 (17.9)	17.5 (19.4)	18.7 (22.2)	18.8 (19.4)	0.09	0.10	0.01
41%–80%	17.8 (20.5)	20.6 (24.7)	20.8 (25.1)	21.7 (21.6)	0.01	0.07	0.06
> 80%	20.2 (23.9)	22.0 (25.6)	19.0 (19.7)	22.4 (23.9)	0.19	0.02	0.22

Abbreviations: CSMS = combined symptom‐medication score, IQR = interquartile range, VAS = visual analogue scale. Cells in orange indicate a small but meaningful effect size (between 0.2 and 0.5) and cells in green indicate a large effect size (higher than 0.8).

^a^ Group corresponding to patients using any kind of rhinitis medication and, therefore, not corresponding to the sum of weeks and users using oral antihistamines, intranasal corticosteroids and azelastine‐fluticasone.

Users with and without self‐reported asthma displayed a similar adherence to rhinitis medication (Table 3). The only exception was observed for INCS, for which a meaningfully higher adherence was observed for patients with asthma (44.9% weeks with adherence vs. 29.3% in patients without asthma; effect size = 0.32).

**TABLE 3:** Comparison of the adherence to rhinitis medications in patients with and without self‐reported asthma.

	No asthma	Asthma	Effect size
Adherence classes to all rhinitis medications^a^–*N* weeks (%)			
0%	1524 (31.0)	838 (25.5)	0.12
1%–40%	540 (11.0)	298 (9.1)	0.05
41%–80%	562 (11.4)	333 (10.1)	0.04
> 80%	2296 (46.6)	1821 (55.3)	0.17
Adherence classes to oral antihistamines–*N* weeks (%)			
0%	1818 (40.6)	1174 (40.1)	0.01
1%–40%	535 (11.9)	281 (9.6)	0.07
41%–80%	464 (10.4)	284 (9.7)	0.02
> 80%	1663 (37.1)	1186 (40.5)	0.07
Adherence classes to intranasal corticosteroids—*N* weeks (%)			
0%	1218 (52.4)	644 (36.4)	0.32
1%–40%	202 (8.7)	154 (8.7)	0
41%–80%	222 (9.6)	176 (10.0)	0.01
> 80%	681 (29.3)	793 (44.9)	0.32
Adherence classes to azelastine‐fluticasone–*N* weeks (%)			
0%	620 (51.1)	615 (54.9)	0.08
1%–40%	90 (7.4)	86 (7.7)	0.01
41%–80%	106 (8.7)	76 (6.8)	0.07
> 80%	398 (32.8)	343 (30.6)	0.05

^a^ Group corresponding to patients using any kind of rhinitis medication and, therefore, not corresponding to the sum of weeks and users using oral antihistamines, intranasal corticosteroids and azelastine‐fluticasone. Cells in orange indicate a small but meaningful effect size (between 0.2 and 0.5).

### 3.3 Medication Adherence and Reported Rhinitis Control

**FIGURE 1:**
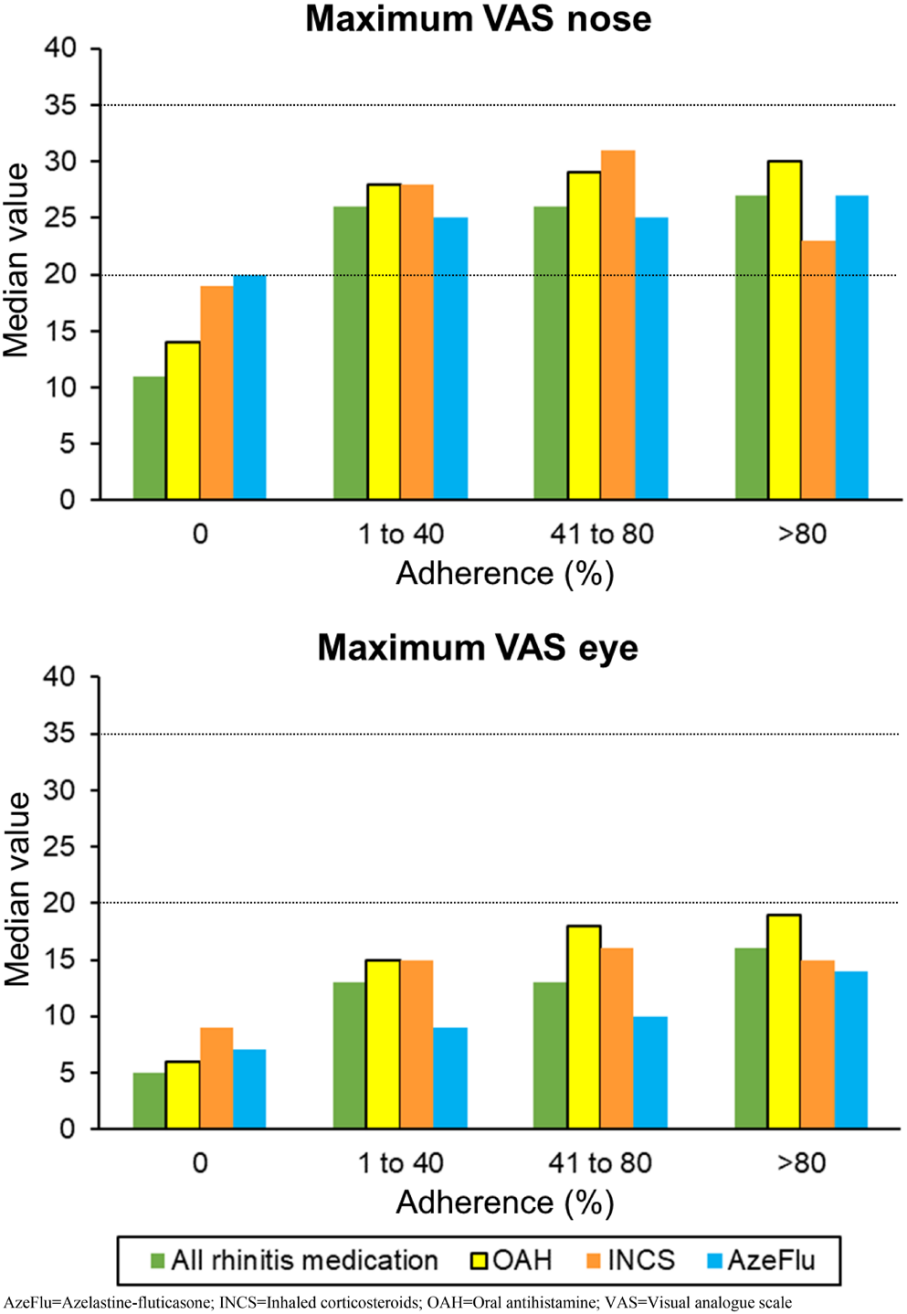
Median maximal levels of VAS nose and VAS eye according to the drug class and to the adherence level.

Overall, median and maximal VAS and CSMS levels increased from no adherence to full adherence. That is, higher levels of adherence were associated with higher VAS and CSMS levels. The only exception concerned INCS: adherence to this drug class was often associated with lower maximal VAS and CSMS levels than partial adherence (Table 2; Figure 1). Similar patterns were observed when considering individual INCS, namely fluticasone furoate and mometasone (Table 4). In fact, for weeks of partial adherence, AzeFlu was associated with lower reported maximal VAS nose levels than INCS, while for weeks with high adherence, these differences were smaller. For ocular symptoms, AzeFlu was associated with lower VAS eye levels than fluticasone furoate or mometasone for all adherence classes.

**TABLE 4:** Adherence and frequency of not well controlled days (Visual Analogue Scale [VAS] > 20) in patients who reported ever use of fluticasone furoate, mometasone and azelastine‐fluticasone (AzeFlu) in complete weeks (weeks with all days of MASK‐air reporting).

	Fluticasone furoate	Mometasone	AzeFlu	Effect size		
				Fluticasone furoate vs. mometasone	Fluticasone furoate vs. AzeFlu	Mometasone vs. AzeFlu
Adherence classes–*N* weeks (%)						
0%	686 (56.0)	1166 (48.5)	1235 (52.9)	0.15	0.06	0.09
1–%40%	94 (7.7)	181 (7.5)	176 (7.5)	0.01	0.01	0
41%–80%	122 (10.0)	206 (8.6)	182 (7.8)	0.05	0.08	0.03
> 80%	324 (26.4)	852 (35.4)	741 (31.7)	0.20	0.12	0.08
Weekly median VAS nose per adherence class–median (IQR)						
0%	9 (21)	12 (19)	11 (19)	0.23	0.14	0.07
1%–40%	11 (17)	16 (20)	11 (16)	0.37	0	0.38
41%–80%	13 (23)	19 (23)	14 (20)	0.40	0.09	0.31
> 80%	13 (26)	15 (18)	14 (25)	0.13	0.06	0.07
Weekly maximum VAS nose per adherence class–median (IQR)						
0%	19 (36)	21 (28)	20 (27)	0.09	0.04	0.05
1%–40%	31 (41)	28 (32)	25 (28)	0.12	0.24	0.15
41%–80%	29 (54)	34 (36)	25 (38)	0.20	0.17	0.38
> 80%	24 (41)	24 (28)	27 (37)	0	0.11	0.14
Weekly median VAS eye per adherence class–median (IQR)						
0%	4 (12)	4 (12)	3 (10)	0	0.15	0.15
1%–40%	5 (15)	5 (13)	4 (14)	0	0.15	0.15
41%–80%	7 (21)	5 (20)	4 (14)	0.22	0.35	0.15
> 80%	7 (25)	6 (14)	5 (15)	0.10	0.22	0.12
Weekly maximum VAS eye per adherence class–median (IQR)						
0%	10 (24)	9 (21)	7 (21)	0.04	0.20	0.17
1%–40%	18 (26)	14 (33)	9 (26)	0.21	0.54	0.34
41%–80%	17 (45)	14 (39)	10 (31)	0.14	0.35	0.23
> 80%	20 (43)	12 (24)	14 (31)	0.37	0.26	0.12
Not well controlled days (VAS nose) per adherence class–*N* (%)						
0%	1310 (27.3)	2543 (31.2)	2436 (28.2)	0.09	0.02	0.07
1%–40%	202 (30.7)	478 (37.7)	386 (31.3)	0.15	0.01	0.13
41%–80%	298 (34.9)	662 (45.9)	433 (34.0)	0.22	0.02	0.24
> 80%	782 (34.5)	2093 (35.1)	2068 (39.9)	0.01	0.11	0.10
Not well controlled days (VAS eye) per adherence class–*N* (%)						
0%	781 (16.3)	1387 (17.0)	1118 (13.7)	0.02	0.07	0.09
1%–40%	132 (20.1)	261 (20.6)	225 (18.3)	0.01	0.05	0.06
41%–80%	233 (27.3)	363 (25.2)	259 (20.3)	0.05	0.16	0.12
> 80%	702 (31.0)	1193 (20.0)	1099 (21.2)	0.25	0.22	0.03
Co‐medication days per adherence class–*N* (%^a^)						
1%–40%	80 (62.0)	252 (54.8)	131 (52.0)	0.15	0.20	0.06
41%–80%	316 (66.1)	826 (49.6)	341 (46.4)	0.34	0.40	0.06
> 80%	1342 (60.7)	5846 (50.3)	3117 (61.4)	0.21	0.01	0.22

Abbreviations: CSMS = combined symptom‐medication score, IQR = interquartile range, VAS = visual analogue scale. Cells in orange indicate a small but meaningful effect size (between 0.2 and 0.5) and cells in yellow indicate a moderate effect size (between 0.5 and 0.8).

^a^ Percentage of the days on which each medication class is used. Not possible to calculate for the 0% adherence class, as, for that class, the respective rhinitis medications are not being used on any weekly day.

A higher proportion of uncontrolled days was observed in weeks with higher adherence. In fact, for weeks with no adherence, the proportion of not well controlled days on nasal symptoms (VAS nose > 20/100) ranged between 16.9% and 29.4%, while for ocular symptoms the proportion ranged from 10.1% to 18.0%. On the other hand, when considering weeks with adherence, the proportion of not well controlled days ranged from 34.2% to 42.1% for VAS nose and from 21.2% to 27.9% for VAS eye (Table 5). In mixed‐effects regression models, a higher number of not well controlled days (both on nasal and ocular symptoms) was associated with a higher weekly percentage of use of rhinitis medication (Table 6).

**TABLE 5:** Frequency of not well controlled days (Visual Analogue Scale [VAS] > 20) and of co‐medication days per medication adherence class in weeks with all days of MASK‐air reporting.

	All rhinitis medications^a^	OAH	INCS	AzeFlu	Effect size		
					OAH vs. INCS	OAH vs. AzeFlu	INCS vs. AzeFlu
Not well controlled days (VAS nose) per adherence class–*N* (%)							
0%	2800 (16.9)	4177 (19.9)	3836 (29.4)	2436 (28.2)	0.22	0.19	0.03
1%–40%	1656 (28.2)	1797 (31.5)	871 (35.0)	386 (31.3)	0.07	0.01	0.08
41%–80%	1955 (31.2)	1842 (35.2)	1114 (40.0)	433 (34.0)	0.10	0.03	0.12
> 80%	11,309 (39.2)	8404 (42.1)	3531 (34.2)	2068 (39.9)	0.16	0.04	0.12
Not well controlled days (VAS eye) per adherence class–*N* (%)							
0%	1670 (10.1)	2488 (11.9)	2347 (18.0)	1118 (13.7)	0.17	0.05	0.12
1%–40%	946 (16.1)	965 (16.9)	477 (19.1)	225 (18.3)	0.06	0.04	0.02
41%–80%	1192 (19.0)	1283 (24.5)	682 (24.5)	259 (20.3)	0	0.10	0.10
> 80%	7394 (25.7)	5556 (27.9)	2476 (24.0)	1099 (21.2)	0.09	0.16	0.07
Co‐medication days per adherence class–*N* (%^b^)							
1%–40%	168 (14.1)	332 (29.5)	310 (61.8)	131 (52.0)	0.66	0.46	0.20
41%–80%	712 (19.6)	1143 (37.9)	961 (60.2)	341 (46.4)	0.45	0.17	0.28
> 80%	11,638 (41.2)	10,011 (51.4)	5737 (56.8)	3117 (61.4)	0.11	0.20	0.09

Abbreviations: AzeFlu = Azelastine‐fluticasone, INCS = intranasal corticosteroids, OAH = oral antihistamines. Cells in orange indicate a small but meaningful effect size (between 0.2 and 0.5) and cells in yellow indicate a moderate effect size (between 0.5 and 0.8).

^a^ Group corresponding to patients using any kind of rhinitis medication and, therefore, not corresponding to the sum of weeks and users using oral antihistamines, intranasal corticosteroids and azelastine‐fluticasone.

^b^ Percentage of the days on which each medication class is used. Not possible to calculate for the 0% adherence class, as, for that class, the respective rhinitis medications are not being used on any weekly day.

**TABLE 6:** Results of multivariable regression models assessing the association between the number of weekly uncontrolled days (assessed with VAS nose and VAS eye) and the percentage of weekly days using rhinitis medications. Results are presented as regression coefficients (95% CI) [*p*‐value].

	All rhinitis medications	Oral antihistamines	Intranasal corticosteroids	Azelastine‐fluticasone
*N* uncontrolled days				
VAS nose	2.8 (2.4;3.2) [< 0.001]	3.0 (2.5;3.4) [< 0.001]	0.9 (0.3;1.4) [0.003]	1.4 (0.6;2.3) [< 0.001]
VAS eye	2.2 (1.8;2.7) [< 0.001]	2.3 (1.8;2.8) [< 0.001]	2.2 (1.5;2.9) [< 0.001]	0.4 (−0.7;1.4) [0.517]

*Note:* As an example of interpretation, each increase of one uncontrolled day in VAS nose levels is associated with an average increase of 2.8% points in the frequency of weekly use of rhinitis medication.

Abbreviations: CI = confidence interval, VAS = visual analogue scale.

When considering weeks with adherence, 41.2% of the days with rhinitis medication use (of any type) involved the use of co‐medication. This percentage was 51.4% for OAH, 56.8% for INCS and 61.4% for AzeFlu (Table 5). For weeks with partial adherence, except for INCS, drugs were used in co‐medication in lower proportions of days: 35.6% for OAH and 47.8% for AzeFlu.

Consistent results were observed in sensitivity analyses considering data from weeks with 6 or 7 days, or months with at most four missing days (Tables [Link], [Link]).

### 3.4 Patient Variability in Adherence Levels

**FIGURE 2:**
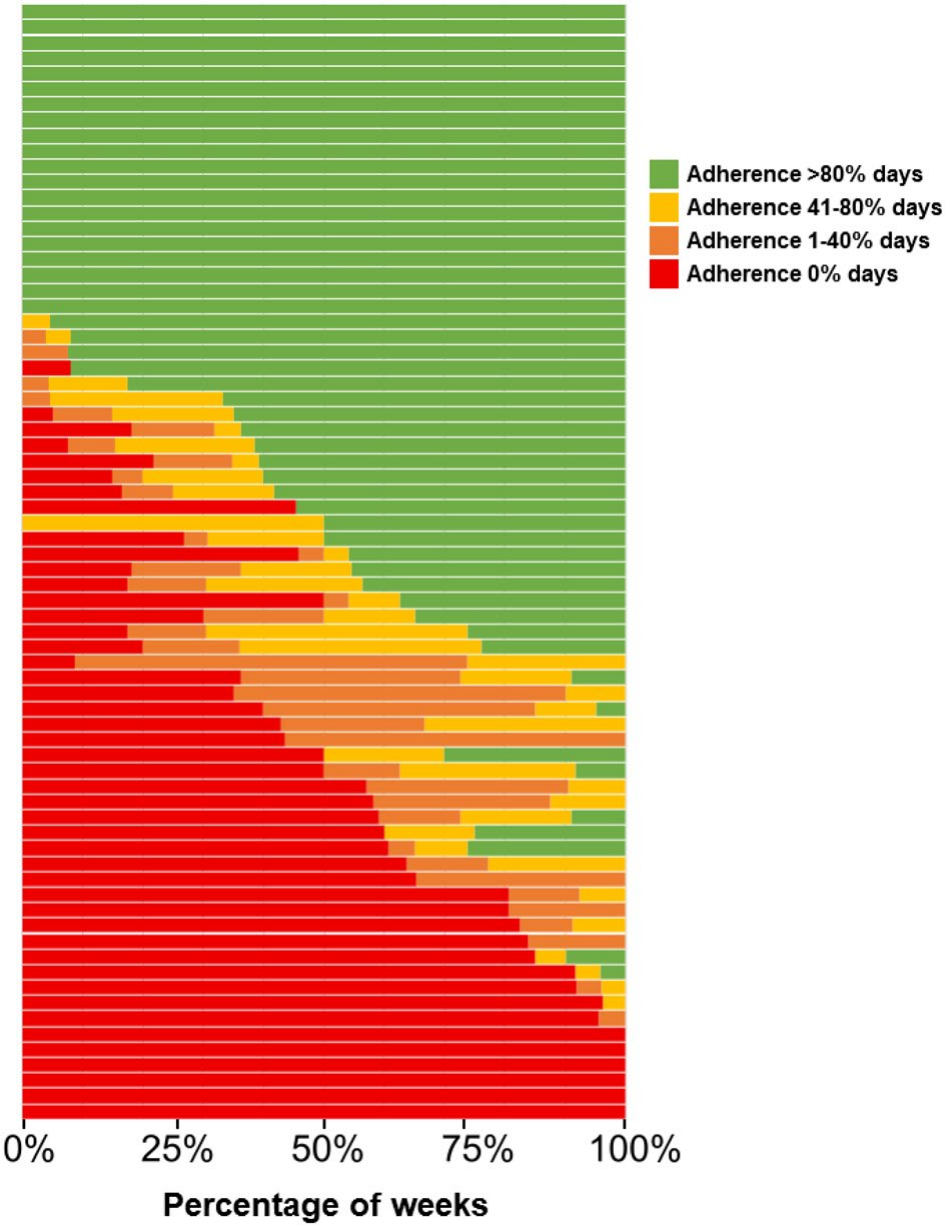
Frequency of weeks per adherence level for each patient from January to July. Each row corresponds to a patient from January to July.

There were 72 patients reporting 6 or 7 days of MASK‐air data for more than 80% of the weeks from January to June. Of those, 25 patients (35%) reported full adherence in all or almost all weeks (Figure 2). On the other hand, 15 patients (21%) never reported medication use or only taking it in exceptional weeks. The remaining 32 patients (44%) displayed a more variable adherence pattern.

## 4 Discussion

In this large allergic rhinitis study using mHealth real‐world data and the validated MASK‐air app, we found a high level of adherence to rhinitis medications. While medication adherence was overall similar across the compared medication classes, there were differences associated with disease control or medication use when considering different medications and different adherence levels.

### 4.1 Limitations and Strengths

As in any mHealth study, there are several limitations to be considered [15]. In fact, selection biases are likely, as MASK‐air users are not representative of the general population with rhinitis (being younger, more able to use digital tools and potentially with higher access to care). MASK‐air users included in the present study may have adherence patterns different from those included in the full dataset. However, although the former are more likely to report asthma or conjunctivitis, median VAS and CSMS levels are similar among users reporting larger volumes of data in comparison to the full MASK‐air dataset (Table S3). In this study, we assessed users reporting 6–7 days a week or over 26 days per month. This probably represents another selection bias, not only because days when patients report data on MASK‐air may be systematically different from the remainder (e.g., days with more severe symptoms), but also because medication adherence may be lower in users with incomplete reporting, as found previously [11]. That is, users who tend to be more adherent to the mHealth app MASK‐air may tend to be more adherent to medication as well (each included patient in this study reported an average of 122 days of MASK‐air compared to 10 days for the remaining users). Nevertheless, an effect of MASK‐air use on medication adherence may not be excluded—it is possible that patients who start using the app may become more self‐aware of their allergic rhinitis and, as a result, start using their medication more regularly. In this context, future studies may assess whether improvement of app adherence could increase medication adherence.

There may also be some information bias, as it is possible that patients do not always report the medications used when answering to the daily monitoring questionnaire. Finally, although MASK‐air was used to assess medication adherence, in the literature, there is no clear definition as to what is considered “adherent” or “non‐adherent” in terms of app usage. In fact, there is no gold‐standard for measuring adherence to medication (particularly in the mHealth context). There are mainly direct and indirect methods. Direct adherence calculation methods include those based on tablet counts and electronic monitoring by medication containers. However, using MASK‐air, adherence measures cannot be directly calculated using a classical method. In the present paper, we used an indirect approach to estimate the proportion of days covered, corresponding to the ratio of days on which medication was reported to be used to days in a given time interval.

This study also has important strengths. It assesses MASK‐air data longitudinally and the sample size is large. Additional strengths concern the assessment of (i) periods with no missing data (allowing to fully assess medication adherence) and (ii) clinically relevant outcomes including VAS with a high validity, reliability and responsiveness [16] and a daily electronic combined symptom‐medication score (CSMS) [12]. Furthermore, we used data directly provided by the patients allowing us to overcome information biases in data collection or provision resulting from researchers' or participants' expectations about a study. Finally, although most of the clinically relevant results have a small effect size, they were consistent throughout the study.

### 4.2 Interpretation

Medication adherence in RCTs is high but does not reflect real‐life situations. An alternative measurement of adherence in a real‐life setting is therefore required. Few studies reported adherence to rhinitis medications in real‐life contexts and adherence levels were far lower than those required by RCTs [17, 18]. In a previous MASK‐air observational cross‐sectional study, where adherence to rhinitis medication was assessed in users reporting not necessarily consecutive days, 11.3% were adherent (medication use ≥ 70% days), 4.2% were partly adherent, 15% were switchers and 69% were non‐adherent to medications [11]. However, the present study was conducted in a different way. It assessed consecutive periods of MASK‐air reporting (rather than any user with a certain amount of data irrespective of the timing and, therefore, rendering it less prone to biases in adherence calculation), comparing the different medication classes and taking into account the association between adherence and reported symptoms. Nevertheless, it seems that having higher adherence to the app may be associated with increased medication adherence (Hawthorne effect) [19].

The three medications had relatively similar patterns of adherence and there were no meaningful differences when comparing users with versus without asthma. Overall, most weeks or months displayed either no adherence (i.e., treatment‐free weeks, 28.8%) or full adherence (50.1%).

As previously found in MASK‐air, when patients were better controlled, they did not report any treatment [5, 20, 21, 22]. However, in treatment‐free weeks, the median or maximal levels of VAS were consistently lower for OAH than for INCS or AzeFlu. These data indicate that users reporting OAH have lower baseline symptoms in their treatment‐free weeks than those reporting INCS or AzeFlu. These data suggest that in real‐life, patients with mild intermittent symptoms tend more often to be treated with OAH (in line with the fact that, in many countries, OAH are dispensed over‐the‐counter). This is the first MASK‐air study to show these differences.

By contrast, in full adherence weeks, users reporting OAH tended to be less well controlled than those reporting INCS or AzeFlu. On the other hand, in weeks with partial adherence, patients under AzeFlu reported lower maximal nasal and ocular VAS levels than those under INCS. These data may possibly suggest that INCS and AzeFlu may differ depending on adherence levels.

Differences in reported symptoms according to adherence levels were also found for intranasal medications. Although we could not consider all individual medications (since we limited them to those with at least 1000 weeks of data), we found that for partial adherence weeks, fluticasone furoate and mometasone were associated with a nasal VAS level usually higher than AzeFlu. For an adherence level of over 80%, the three medications had similar VAS levels. AzeFlu was associated with lower VAS eye levels than fluticasone furoate or mometasone. These data have never been reported before and raise the hypothesis that the effect of medications might differ according to medication adherence. However, this needs to be confirmed with more adequate causal inference approaches. These data, associated with a recent meta‐analysis [23], will have an impact on ARIA 2024 [24].

Co‐medication was common at all adherence levels and a higher frequency of co‐medication was observed in weeks with adherence. For partial adherence, AzeFlu users reported less co‐medication than INCS users. However, for full adherence periods, there were no meaningful differences. It has been consistently found in MASK‐air that INCS‐OAH co‐medication was more associated with less well controlled days than INCS alone [5, 20, 21, 22]. The differences between both groups reached the minimal important difference [25]. On the other hand, OAH users reported less co‐medication than INCS users or AzeFlu users, possibly reflecting the wider use of OAH in less severe patients not requiring co‐medication.

Although the results of the present study are highly consistent in sensitivity analyses (weeks with 7 days of reporting, weeks with 6–7 days of reporting and months with at most 4 days missing), mHealth data from observational studies are only hypothesis‐generating and should be confirmed in proper studies. However, current RCTs cannot be used since patients with an adherence of under 70% are excluded. These data may have a relevant impact on guideline generation, stressing the importance of adherence levels, and indicating that a non‐negligible amount of patients do not use medication every day but rather on an as‐needed basis. Comparing the chronic versus as‐needed use of medication is paramount and, if as‐needed use starts to be recommended, the term ‘adherence’ may cease to be the most adequate in relation to medication use patterns.

## 5 Conclusion

In this study, we used real‐world mHealth data to assess adherence to rhinitis medication. (i) We observed a high adherence to medication (with no meaningful differences across medication classes) possibly associated with selection biases as we studied patients adherent to the app. This study suggests that higher MASK‐air app use is associated with increased medication adherence. (ii) We found meaningful differences in symptom control and co‐medication use when comparing INCS and other medication classes depending on adherence levels. (iii) The control of rhinitis decreased with increasing adherence confirming previous MASK‐air studies suggesting that patients often use medication when they have symptoms. These results point to the need for assessing the effectiveness of rhinitis medication in scenarios of suboptimal adherence and/or *pro re nata* use.

## Author Contributions

Jean Bousquet, Wienczyslawa Czarlewski, Torsten Zuberbier, Joao A Fonseca and Bernardo Sousa‐Pinto participated in the study design. Bernardo Sousa‐Pinto, Elísio M. Costa and Rafael José Vieira participated in data analyses. Jean Bousquet, Elísio M. Costa and Bernardo Sousa‐Pinto participated in writing the original draft of the paper. The remaining members participated in data retrieval and in the critical revision of the manuscript.

## Ethics Statement

MASK‐air complies with the General Data Protection Regulation. Although an IRB is not needed for observational studies, the use of MASK‐air data for research purposes has been approved by an independent review board (Köln‐Bonn, Germany). Users consented to having their data analysed for scientific purposes in terms of use of the app. All data wase provided by users anonymously.

## Conflicts of Interest

JB reports personal fees from Cipla, Menarini, Mylan, Novartis, Purina, Sanofi‐Aventis, Teva, Noucor, other from KYomed‐Innov, other from Mask‐air‐SAS, outside the submitted work. ICO reports other from Sanofi‐Aventis, outside the submitted work. PD reports personal fees and non‐financial support from Astra Zeneca, personal fees from Chiesi, personal fees and non‐financial support from Boehringer Ingelheim, personal fees from GlaxoSmithKline, personal fees from Menarini, personal fees and non‐financial support from Stallergenes, personal fees and non‐financial support from ALK Abello, outside the submitted work. MM reports other from ASTRA ZENECA, other from SANOFI AVENTIS, other from PFIZER, personal fees from TAKEDA, other from CHIESI, outside the submitted work. OP reports grants and personal fees from ALK‐Abelló, grants and personal fees from Allergopharma, grants and personal fees from Stallergenes Greer, grants and personal fees from HAL Allergy Holding B.V./HAL Allergie GmbH, grants from Bencard Allergie GmbH/Allergy Therapeutics, grants from Lofarma, grants and personal fees from ASIT Biotech Tools S.A., grants and personal fees from Laboratorios LETI/LETI Pharma, grants and personal fees from GlaxoSmithKline, personal fees from ROXALL Medizin, personal fees from Novartis, grants and personal fees from Sanofi‐Aventis and Sanofi‐Genzyme, personal fees from Med Update Europe GmbH, personal fees from streamedup! GmbH, grants from Pohl‐Boskamp, grants from Inmunotek S.L., personal fees from John Wiley and Sons, AS, personal fees from Paul‐Martini‐Stiftung (PMS), personal fees from Regeneron Pharmaceuticals Inc., personal fees from RG Aerztefortbildung, personal fees from Institut für Disease Management, personal fees from Springer GmbH, grants and personal fees from AstraZeneca, personal fees from IQVIA Commercial, personal fees from Ingress Health, personal fees from Wort&Bild Verlag, personal fees from Verlag ME, personal fees from Procter&Gamble, personal fees from ALTAMIRA, personal fees from Meinhardt Congress GmbH, personal fees from Deutsche Forschungsgemeinschaft, personal fees from Thieme, grants from Deutsche AllergieLiga e.V., personal fees from AeDA, personal fees from Alfried‐Krupp Krankenhaus, personal fees from Red Maple Trials Inc., personal fees from Königlich Dänisches Generalkonsulat, personal fees from Medizinische Hochschule Hannover, personal fees from ECM Expro&Conference Management, personal fees from Technical University Dresden, personal fees from Lilly, personal fees from Paul Ehrlich Institut, personal fees from Japanese Society of Allergy, personal fees from Forum für Medizinische Fortbildung, from Dustri‐Verlag, outside the submitted work; and member of EAACI Excom, member of ext. board of directors DGAKI; coordinator, main‐ or co‐author of different position papers and guidelines in rhinology, allergology and allergen‐immunotherapy. VK reports non‐financial support from NORAMEDA, non‐financial support from DIMUNA, non‐financial support from Berlin CHemie Menarini, outside the submitted work. TH reports personal fees from Orion Pharma, outside the submitted work. DLL reports personal fees from ALK, Astrazeneca national and global, Bayer, Chiesi, Grunenthal, Grin, GSK national and global, Viatris, Menarini, MSD, Novartis, Pfizer, Sanofi, Siegfried, UCB, Carnot, grants from Abbvie, Bayer, Lilly, Sanofi, Astrazeneca, Pfizer, Novartis, Circassia, UCB, GSK., outside the submitted work. NP reports grants from Capricare, personal fees from Nestle, personal fees from Numil, personal fees from Vianex, personal fees from REG, outside the submitted work. JCI reports personal fees from Laboratorios Casasco, personal fees from Bago Bolivia, personal fees from Abbott Ecuador, personal fees from Faes Farma, outside the submitted work. LTB reports personal fees from AstraZeneca, personal fees from LETI Laboratories, personal fees from Sanofi, outside the submitted work. LC reports personal fees from Novartis, personal fees from Astra Zeneca, personal fees from GSK, personal fees from ALK, personal fees from Thermofisher, personal fees from Sanofi, outside the submitted work. RL reports grants and personal fees from GSK, grants and personal fees from AZ, grants from Chiesi, outside the submitted work. HK reports personal fees from Sanofi, personal fees from Berlin‐Chemie, personal fees from Viatris/Mylan, outside the submitted work. BS reports personal fees from Polpharma, personal fees from Viatris, grants and personal fees from AstraZeneca, personal fees from TEVA, personal fees from patient ombudsman, personal fees from Polish Allergology Society, grants from GSK, personal fees from ADAMED, outside the submitted work. MZ reports personal fees from Takeda, outside the submitted work. IA reports personal fees from Abbott, personal fees from Bayer, personal fees from Bial, personal fees from Eurodrug, personal fees from Faes Farma, personal fees from Gebro, personal fees from Menarini, personal fees from MSD, personal fees from Roxall, personal fees from Sanofi, outside the submitted work. JS reports grants and personal fees from SANOFI, personal fees from GSK, personal fees from NOVARTIS, personal fees from ASTRA ZENECA, personal fees from MUNDIPHARMA, personal fees from FAES FARMA, outside the submitted work. STS reports grants and other from Sanofi, grants and other from GSK, other from Clario, other from Orion Pharma, other from ALK‐Abelló, other from Roche, other from AstraZeneca, outside the submitted work. TZ reports grants and personal fees from Novartis, grants and personal fees from Henkel, personal fees from Bayer, personal fees from FAES, personal fees from Astra Zeneca, personal fees from AbbVie, personal fees from ALK, personal fees from Almirall, personal fees from Astellas, personal fees from Bayer, personal fees from Bencard, personal fees from Berlin Chemie, personal fees from FAES, personal fees from Hal, personal fees from Leti, personal fees from Mesa, personal fees from Menarini, personal fees from Merck, personal fees from MSD, personal fees from Novartis, personal fees from Pfizer, personal fees from Sanofi, personal fees from Stallergenes, personal fees from Takeda, personal fees from Teva, personal fees from UCB, personal fees from Henkel, personal fees from Kryolan, personal fees from L'Oreal, outside the submitted work; and Organisational affiliations: Committee member: WHO‐Initiative ‘Allergic Rhinitis and Its Impact on Asthma’ (ARIA); Member of the Board: German Society for Allergy and Clinical Immunology (DGAKI); Head: European Centre for Allergy Research Foundation (ECARF); President: Global Allergy and Asthma European Network (GA2LEN); Member: Committee on Allergy Diagnosis and Molecular Allergology, World Allergy Organisation (WAO). AC reports personal fees from AstraZeneca, personal fees from Boehringer‐Ingelheim, personal fees from Chiesi, personal fees from GSK, personal fees from Sanofi, personal fees from Eurofarma, personal fees from Novartis, personal fees from Crossject, personal fees from Glennmark, personal fees from Abdi Ibrahim, personal fees from Mylan, personal fees from Farmoquimica, personal fees from Ache, outside the submitted work. PK reports personal fees from Adamed, personal fees and other from Berlin Chemie Menarini, personal fees from Boehringer Ingelheim, personal fees from AstraZeneca, personal fees from Celon Pharma, personal fees from FAES, personal fees from Novartis, personal fees from Polpharma, personal fees from GSK, personal fees from Sanofi, personal fees from Teva, personal fees from Zentiva, outside the submitted work. The other authors have no COI to disclose, outside the submitted work.

## Supporting information


Figure S1.

Figure S2.

Table S1.

Table S2.

Table S3.

Table S4.

Table S5.

Table S6.

## Data Availability

Individual participant data underlying the results reported in this Article can be made available (after de‐identification) between 12 and 36 months after Article publication. These data can be supplied to researchers who provide a methodologically sound proposal. Proposals should be directed to the corresponding author (jean.bousquet@orange.fr). We made every effort to follow the EU General Data Protection Regulation; therefore, we can transfer data only if there is a protocol and an agreement between the owner of the data and the person (or institution) requesting the data. To gain access, data requestors will need to sign a data access agreement.
