# Supplementary material for: Adherence to Treatment in Allergic Rhinitis During the Pollen Season in Europe: A MASK‐air Study
Source: Clin Exp Allergy. 2025 Feb 16;55(3):226–38. doi: 10.1111/cea.70004 (PMC11908838; doi:10.1111/cea.70004)
Supplement: Supplementary file 1 — Figure S1. [file CEA-55-226-s008.pdf]

Supplementary Figure 1. Flow chart of patient selection

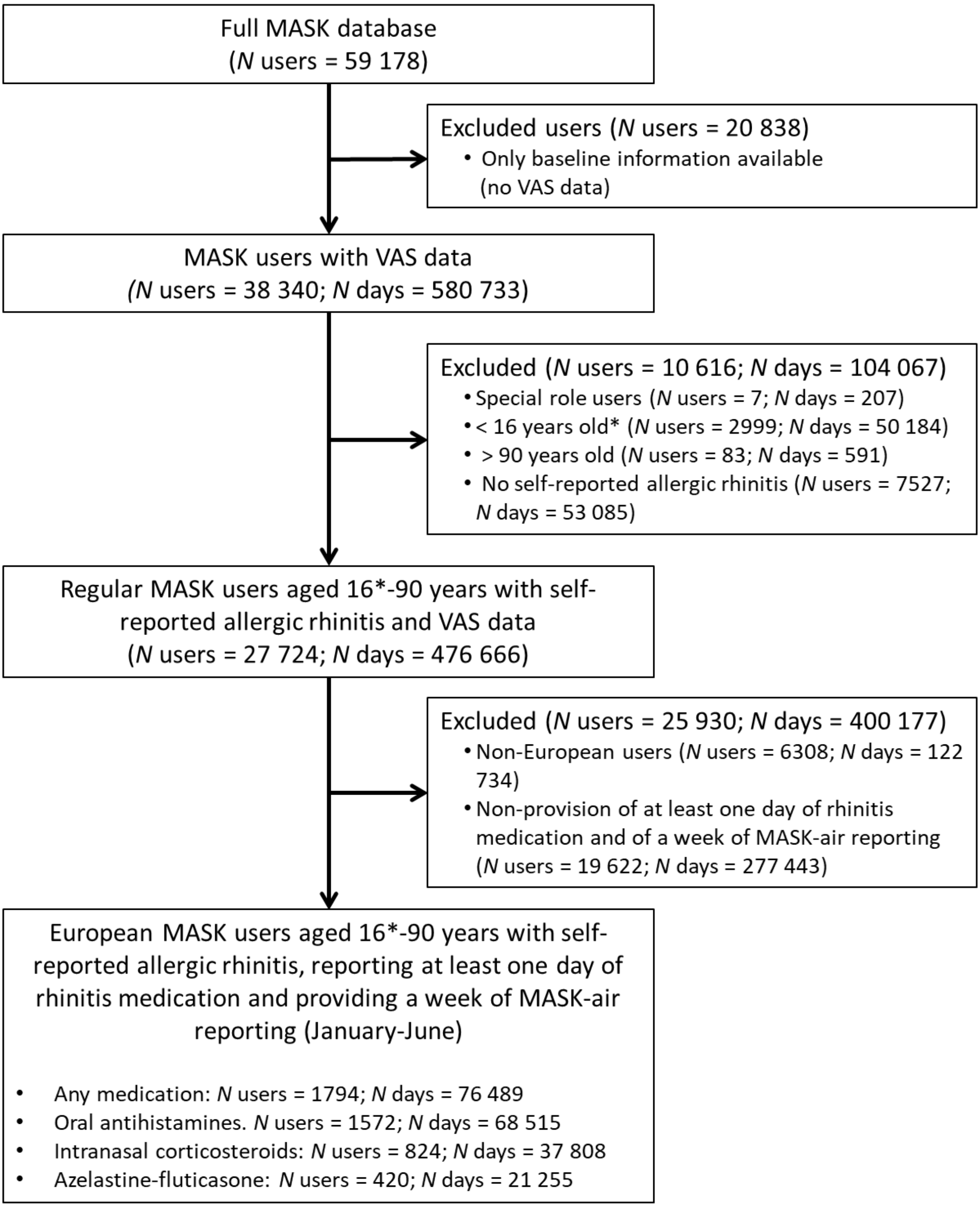

*Or lower (not below 13 years old) for countries where the digital age of consent is lower
