# Supplementary material for: Adherence to Treatment in Allergic Rhinitis During the Pollen Season in Europe: A MASK‐air Study
Source: Clin Exp Allergy. 2025 Feb 16;55(3):226–38. doi: 10.1111/cea.70004 (PMC11908838; doi:10.1111/cea.70004)
Supplement: Supplementary file 2 — Figure S2. [file CEA-55-226-s004.pdf]

Supplementary Figure 2. Percentage of reported weeks according to medication adherence levels

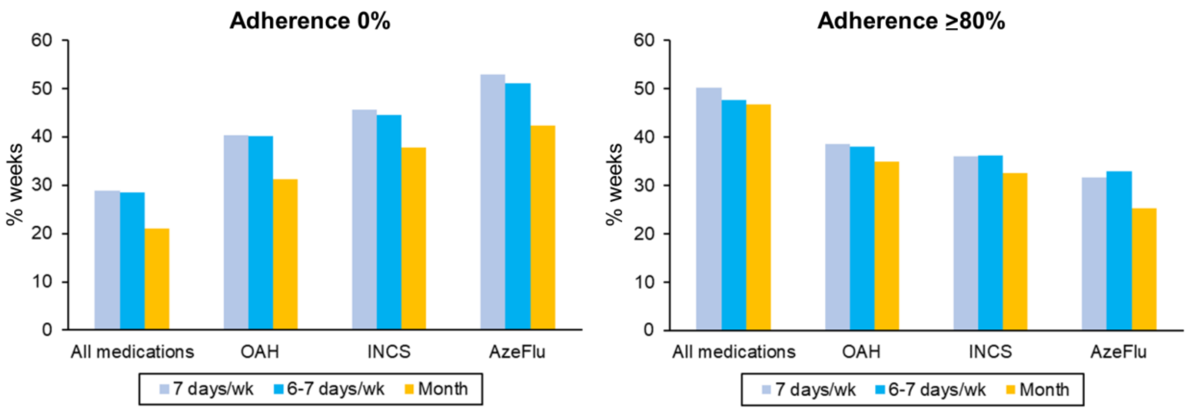

AzeFlu=Azelastine-fluticasone; INCS=Inhaled corticosteroids; OAH=Oral antihistamine; wk=Week
