# Supplementary material for: Adherence to Treatment in Allergic Rhinitis During the Pollen Season in Europe: A MASK‐air Study
Source: Clin Exp Allergy. 2025 Feb 16;55(3):226–38. doi: 10.1111/cea.70004 (PMC11908838; doi:10.1111/cea.70004)
Supplement: Supplementary file 3 — Table S1. [file CEA-55-226-s007.pdf]

Supplementary Table 1. Visual analogue scales (VASs) used for symptoms daily monitoring in MASK-air®

Scale	Question
**VAS Global allergy symptoms**	"Overall how much are your allergic symptoms bothering you today?"
**VAS Nose**	"How much are your nose symptoms bothering you today?"
**VAS Eyes**	"How much are your eye symptoms bothering you today?"
**VAS Asthma**	"How much are your asthma symptoms bothering you today?"
