# Supplementary material for: Adherence to Treatment in Allergic Rhinitis During the Pollen Season in Europe: A MASK‐air Study
Source: Clin Exp Allergy. 2025 Feb 16;55(3):226–38. doi: 10.1111/cea.70004 (PMC11908838; doi:10.1111/cea.70004)
Supplement: Supplementary file 4 — Table S2. [file CEA-55-226-s006.pdf]

**Supplementary Table 2. Adherence in patients who reported ever use of rhinitis medication in weeks with 6 or 7 days of MASK-air® reporting**

| | All rhinitis medications [a] | Oral antihistamines | Intranasal corticosteroids | Azelastine-fluticasone |
|---|---|---|---|---|
| Complete weeks of MASK-air® reporting | | | | |
| Austria | 180 (2.2) | 154 (2.1) | 103 (2.5) | 87 (3.7) |
| Belgium | 49 (0.6) | 44 (0.6) | 31 (0.8) | 0 |
| Czech Republic | 178 (2.2) | 174 (2.4) | 71 (1.7) | 1 (0.1) |
| Denmark | 42 (0.5) | 41 (0.6) | 15 (0.4) | 0 |
| Finland | 224 (2.7) | 224 (3.0) | 161 (3.9) | 159 (6.8) |
| France | 753 (9.2) | 741 (10.0) | 186 (4.6) | 218 (9.3) |
| Germany | 1298 (15.8) | 1115 (15.1) | 658 (16.1) | 313 (13.4) |
| Great Britain | 139 (1.7) | 56 (0.8) | 120 (2.9) | 10 (0.4) |
| Greece | 270 (3.3) | 164 (2.2) | 131 (3.2) | 168 (7.2) |
| Hungary | 15 (0.2) | 10 (0.1) | 7 (0.2) | 7 (0.3) |
| Italy | 1135 (13.8) | 1007 (13.6) | 525 (12.8) | 292 (12.5) |
| Lithuania | 1502 (18.3) | 1367 (18.5) | 813 (19.9) | 655 (28.1) |
| Netherlands | 264 (3.2) | 259 (3.5) | 85 (2.1) | 24 (1.0) |
| Poland | 557 (6.8) | 555 (7.5) | 198 (4.8) | 196 (8.4) |
| Portugal | 480 (5.9) | 445 (6.0) | 365 (8.9) | 48 (2.1) |
| Slovenia | 40 (0.5) | 35 (0.5) | 27 (0.7) | 14 (0.6) |
| Spain | 830 (10.1) | 759 (10.3) | 455 (11.1) | 104 (4.5) |
| Sweden | 42 (0.5) | 42 (0.6) | 34 (0.8) | 8 (0.3) |
| Switzerland | 214 (2.6) | 213 (2.9) | 105 (2.6) | 30 (1.3) |
| Weeks with 6 or 7 days of MASK-air® reporting | | | | |
| Austria | 243 (2.1) | 201 (2.0) | 128 (2.3) | 119 (3.8) |
| Belgium | 68 (0.6) | 59 (0.6) | 43 (0.8) | 0 |
| Czech Republic | 259 (2.3) | 252 (2.5) | 100 (1.8) | 3 (0.1) |
| Denmark | 47 (0.4) | 46 (0.5) | 20 (0.4) | 0 |
| Finland | 279 (2.5) | 279 (2.7) | 195 (3.5) | 192 (6.1) |
| France | 1110 (9.8) | 1088 (10.7) | 263 (4.7) | 320 (10.1) |
| Germany | 1730 (15.2) | 1443 (14.2) | 896 (15.9) | 399 (12.6) |
| Great Britain | 191 (1.7) | 97 (1.0) | 161 (2.9) | 16 (0.5) |
| Greece | 361 (3.2) | 219 (2.2) | 175 (3.1) | 214 (6.8) |
| Hungary | 27 (0.2) | 20 (0.2) | 11 (0.2) | 10 (0.3) |
| Italy | 1592 (14.0) | 1408 (13.8) | 741 (13.2) | 388 (12.3) |
| Lithuania | 2057 (18.1) | 1878 (18.4) | 1054 (18.7) | 889 (28.2) |
| Netherlands | 366 (3.2) | 355 (3.5) | 119 (2.1) | 36 (1.1) |
| Poland | 807 (7.1) | 799 (7.8) | 280 (5.0) | 261 (8.3) |
| Portugal | 712 (6.3) | 637 (6.3) | 548 (9.8) | 75 (2.4) |
| Slovenia | 59 (0.5) | 53 (0.5) | 40 (0.7) | 23 (0.7) |
| Spain | 1132 (9.9) | 1017 (10.0) | 675 (12.0) | 153 (4.9) |
| Sweden | 69 (0.6) | 68 (0.7) | 51 (0.9) | 18 (0.6) |
| Switzerland | 280 (2.5) | 274 (2.7) | 123 (2.2) | 40 (1.3) |
| Months with at most 4 missing days of MASK-air® reporting | | | | |
| Austria | 37 (2.9) | 33 (2.8) | 23 (3.5) | 18 (4.7) |
| Belgium | 7 (0.5) | 7 (0.6) | 4 (0.6) | 0 |
| Czech Republic | 26 (2.0) | 25 (2.2) | 9 (1.4) | 0 |
| Denmark | 6 (0.5) | 6 (0.5) | 1 (0.2) | 0 |
| Finland | 37 (2.9) | 37 (3.2) | 29 (4.4) | 30 (7.8) |
| France | 102 (8.0) | 102 (8.8) | 26 (4.0) | 25 (6.5) |
| Germany | 210 (16.4) | 185 (15.9) | 111 (16.9) | 57 (14.9) |
| Great Britain | 22 (1.7) | 4 (0.3) | 22 (3.3) | 0 |
| Greece | 40 (3.1) | 23 (2.0) | 21 (3.2) | 26 (6.8) |
| Hungary | 2 (0.2) | 1 (0.1) | 0 | 1 (0.3) |
| Italy | 176 (13.7) | 156 (13.4) | 73 (11.1) | 49 (12.8) |

| | | | | |
|---|---|---|---|---|
| Lithuania | 247 (19.3) | 222 (19.1) | 143 (21.8) | 114 (29.8) |
| Netherlands | 43 (3.4) | 43 (3.7) | 12 (1.8) | 6 (1.6) |
| Poland | 79 (6.2) | 79 (6.8) | 32 (4.9) | 32 (8.4) |
| Portugal | 63 (4.9) | 61 (5.3) | 45 (6.8) | 4 (1.0) |
| Slovenia | 4 (0.3) | 4 (0.3) | 4 (0.6) | 0 |
| Spain | 142 (11.1) | 133 (11.5) | 76 (11.6) | 13 (3.4) |
| Sweden | 5 (0.4) | 5 (0.4) | 4 (0.6) | 1 (0.3) |
| Switzerland | 35 (2.7) | 35 (3.0) | 22 (3.3) | 7 (1.8) |

[a] Group corresponding to patients using any kind of rhinitis medication and, therefore, not corresponding to the sum of weeks and users using oral antihistamines, intranasal corticosteroids and azelastine-fluticasone
