# Supplementary material for: Adherence to Treatment in Allergic Rhinitis During the Pollen Season in Europe: A MASK‐air Study
Source: Clin Exp Allergy. 2025 Feb 16;55(3):226–38. doi: 10.1111/cea.70004 (PMC11908838; doi:10.1111/cea.70004)
Supplement: Supplementary file 5 — Table S3. [file CEA-55-226-s002.pdf]

Supplementary Table 3. Comparison of MASK-air® users included in this study with those of the full MASK-air® dataset

	Patients using any rhinitis medication			All MASK-air® data on users with rhinitis
	Reporting complete weeks of MASK-air®	Reporting weeks with 6 or 7 days of MASK-air®	Reporting months with at most 4 missing days of MASK-air®	
N users	1361	1794	484	27,724
Females – N (%)	711 (52.2)	945 (52.7)	240 (49.6)	15,915 (57.4)
Age – mean (SD)	38.2 (13.8)	37.9 (14.0)	39.6 (14.0)	35.8 (13.5)
Self-reported asthma – N (%)	542 (39.8)	708 (39.5)	177 (36.6)	8698 (31.4)
Conjunctivitis – N (%)	999 (73.4)	1305 (72.7)	365 (75.4)	15,002 (54.1)
VAS nose – median (IQR)	11 (20)	11 (21)	10 (18)	10 (21)
VAS eye – median (IQR)	4 (14)	4 (15)	3 (12)	4 (13)
CSMS – median (IQR)	9.0 (14.4)	9.3 (15.1)	8.1 (13.1)	8.3 (14.0)

CSMS=Combined symptom-medication score; IQR=Interquartile range; SD=Standard-deviation VAS=Visual analogue scale
