# Supplementary material for: Adherence to Treatment in Allergic Rhinitis During the Pollen Season in Europe: A MASK‐air Study
Source: Clin Exp Allergy. 2025 Feb 16;55(3):226–38. doi: 10.1111/cea.70004 (PMC11908838; doi:10.1111/cea.70004)
Supplement: Supplementary file 6 — Table S4. [file CEA-55-226-s003.pdf]

Supplementary Table 4. Adherence in patients who reported ever use of rhinitis medication in weeks with 6 or 7 days of MASK-air® reporting

	All rhinitis medications [a]	Oral antihistamines	Intranasal corticosteroids	Azelastine-fluticasone
Adherence classes – N weeks (%)				
0%	3242 (28.5)	4089 (40.1)	2504 (44.5)	1612 (51.1)
1-40%	1206 (10.6)	1184 (11.6)	548 (9.7)	255 (8.1)
41-80%	1505 (13.2)	1043 (10.2)	539 (9.6)	252 (8.0)
>80%	5436 (47.7)	3877 (38.0)	2032 (36.1)	1037 (32.9)
Weekly median VAS nose per adherence class – median (IQR)				
0%	5 (14)	6 (16)	10 (21)	11 (19)
1-40%	10 (18)	11 (18)	14 (20)	12 (17)
41-80%	13 (21)	14 (22)	17 (24)	13 (19)
>80%	15 (24)	17 (26)	15 (21)	15 (24)
Weekly maximum VAS nose per adherence class – median (IQR)				
0%	12 (24)	14 (25)	20 (33)	20 (28)
1-40%	26 (35)	28 (35)	30 (36)	26 (28)
41-80%	29 (38)	33 (40)	32 (38)	25 (35)
>80%	28 (37)	31 (38)	25 (34)	28 (37)
Weekly median VAS eye per adherence class – median (IQR)				
0%	0 (7)	1 (9)	3 (13)	3 (10)
1-40%	4 (11)	4 (13)	6 (14)	4 (14)
41-80%	6 (17)	7 (21)	7 (19)	5 (16)
>80%	7 (20)	8 (21)	7 (19)	6 (16)
Weekly maximum VAS eye per adherence class – median (IQR)				
0%	6 (18)	7 (20)	10 (25)	7 (22)
1-40%	14 (27)	16 (28)	16 (31)	13 (26)
41-80%	16 (33)	20 (38)	20 (39)	11 (31)
>80%	17 (35)	20 (38)	16 (32)	15 (30)
Weekly median CSMS per adherence class – median (IQR)				
0%	3.5 (9.5)	5.2 (11.3)	7.1 (13.6)	8.6 (13.1)
1-40%	6.8 (11.3)	8.7 (12.6)	9.7 (13.5)	10.0 (13.6)
41-80%	10.2 (14.0)	11.4 (15.7)	13.1 (16.3)	12.3 (14.2)
>80%	13.1 (16.6)	13.7 (18.0)	13.6 (15.1)	15.1 (16.3)
Weekly maximum CSMS per adherence class – median (IQR)				
0%	7.4 (14.8)	9.5 (16.2)	12.9 (19.5)	13.5 (16.8)
1-40%	17.0 (19.7)	18.7 (20.6)	19.6 (23.3)	20.0 (19.8)
41-80%	19.5 (23.6)	22.1 (25.4)	22.1 (27.2)	21.4 (21.2)
>80%	20.7 (24.3)	22.5 (26.5)	19.9 (21.7)	22.8 (23.1)

CSMS=Combined symptom-medication score; IQR=Interquartile range; VAS=Visual analogue scale; [a] Group corresponding to patients using any kind of rhinitis medication and, therefore, not corresponding to the sum of weeks and users using oral antihistamines, intranasal corticosteroids and azelastine-fluticasone
