# Supplementary material for: Adherence to Treatment in Allergic Rhinitis During the Pollen Season in Europe: A MASK‐air Study
Source: Clin Exp Allergy. 2025 Feb 16;55(3):226–38. doi: 10.1111/cea.70004 (PMC11908838; doi:10.1111/cea.70004)
Supplement: Supplementary file 7 — Table S5. [file CEA-55-226-s005.pdf]

Supplementary Table 5. Adherence in patients who reported ever use of rhinitis medication in months with at most 4 missing days of MASK-air® reporting

	All rhinitis medications [a]	Oral antihistamines	Intranasal corticosteroids	Azelastine-fluticasone
Adherence classes – N months (%)				
0%	270 (21.0)	362 (31.2)	248 (37.7)	162 (42.3)
1-40%	261 (20.3)	267 (23.0)	119 (18.1)	86 (22.5)
41-80%	152 (11.8)	127 (10.9)	76 (11.6)	38 (9.9)
>80%	600 (46.8)	405 (34.9)	214 (32.6)	97 (25.3)
Monthly median VAS nose per adherence class – median (IQR)				
0%	3 (10)	4 (13)	10 (22)	11 (20)
1-40%	8 (15)	8 (16)	9 (16)	15 (18)
41-80%	12 (17)	13 (18)	17 (16)	12 (18)
>80%	14 (21)	16 (24)	13 (15)	15 (28)
Monthly maximum VAS nose per adherence class – median (IQR)				
0%	17 (30)	19 (29)	27 (39)	24 (32)
1-40%	32 (34)	36 (33)	31 (30)	37 (36)
41-80%	33 (32)	34 (46)	40 (30)	37 (28)
>80%	35 (42)	42 (44)	29 (32)	41 (53)
Monthly median VAS eye per adherence class – median (IQR)				
0%	0 (5)	0 (6)	3 (14)	0 (9)
1-40%	2 (9)	3 (10)	3 (11)	4 (12)
41-80%	3 (12)	5 (16)	5 (19)	1 (8)
>80%	6 (17)	6 (18)	6 (13)	5 (16)
Monthly maximum VAS eye per adherence class – median (IQR)				
0%	8 (21)	9 (22)	14 (34)	8 (27)
1-40%	19 (31)	19 (31)	23 (32)	15 (33)
41-80%	19 (33)	31 (33)	25 (34)	18 (24)
>80%	25 (39)	28 (42)	22 (33)	22 (41)
Monthly median CSMS per adherence class – median (IQR)				
0%	2.3 (6.9)	3.8 (9.2)	6.2 (13.3)	8.5 (14.3)
1-40%	5.7 (10.4)	6.6 (10.6)	6.9 (10.0)	11.6 (13.9)
41-80%	8.0 (9.8)	9.5 (14.6)	12.0 (12.1)	10.7 (11.6)
>80%	12.2 (14.9)	12.5 (15.4)	12.4 (12.1)	17.4 (15.1)
Monthly maximum CSMS per adherence class – median (IQR)				
0%	8.6 (16.1)	11.0 (17.9)	16.2 (22.3)	17.4 (19.5)
1-40%	21.1 (19.6)	22.3 (21.0)	21.6 (24.7)	25.5 (26.1)
41-80%	20.9 (23.0)	25.4 (26.1)	26.4 (19.0)	23.8 (16.4)
>80%	24.6 (26.3)	27.3 (28.5)	21.6 (24.7)	29.7 (30.0)

CSMS=Combined symptom-medication score; IQR=Interquartile range; VAS=Visual analogue scale; [a] Group corresponding to patients using any kind of rhinitis medication and, therefore, not corresponding to the sum of weeks and users using oral antihistamines, intranasal corticosteroids and azelastine-fluticasone
