# Supplementary material for: Adherence to Treatment in Allergic Rhinitis During the Pollen Season in Europe: A MASK‐air Study
Source: Clin Exp Allergy. 2025 Feb 16;55(3):226–38. doi: 10.1111/cea.70004 (PMC11908838; doi:10.1111/cea.70004)
Supplement: Supplementary file 8 — Table S6. [file CEA-55-226-s001.pdf]

Supplementary Table 6. Frequency of not well controlled days (Visual Analogue Scale [VAS]>20) or of co-medication days per adherence class considering weeks with at most one missing day and most with at most four missing days of MASK-air® reporting

	All rhinitis medications [a]	Oral antihistamines	Intranasal corticosteroids	Azelastine-fluticasone
**A. Weeks with at most one missing day (6-7 days of reporting data)**				
Not well controlled days (VAS nose) per adherence class – N (%)				
0%	3851 (17.0)	5666 (20.6)	5048 (29.9)	3084 (28.3)
1-40%	2354 (27.9)	2558 (32.3)	1326 (36.4)	556 (32.6)
41-80%	3461 (32.9)	2648 (37.9)	1502 (41.4)	567 (33.5)
>80%	14,619 (38.4)	11,125 (42.6)	4900 (35.9)	2813 (40.4)
Not well controlled days (VAS eye) per adherence class – N (%)				
0%	2375 (10.5)	3480 (12.7)	3087 (18.3)	1540 (14.1)
1-40%	1372 (16.3)	1475 (18.6)	757 (20.8)	312 (18.3)
41-80%	2177 (20.7)	1858 (26.6)	950 (26.2)	361 (21.3)
>80%	9573 (25.2)	7408 (28.4)	3379 (24.7)	1489 (21.4)
Co-medication days per adherence class – N (% [a])				
1-40%	242 (14.0)	518 (31.5)	461 (59.9)	170 (46.7)
41-80%	1445 (23.3)	1594 (39.3)	1284 (61.1)	474 (48.2)
>80%	14,872 (41.1)	12,889 (50.7)	7622 (57.1)	4022 (59.2)
**B. Months with at most four missing days**				
Not well controlled days (VAS nose) per adherence class – N (%)				
0%	1043 (13.4)	1784 (17.1)	2224 (30.9)	1243 (26.5)
1-40%	1774 (23.5)	2023 (26.3)	908 (26.5)	948 (38.1)
41-80%	1200 (27.4)	1175 (32.1)	823 (37.6)	303 (27.8)
>80%	6480 (37.3)	4733 (40.3)	1839 (29.6)	1205 (43.1)
Not well controlled days (VAS eye) per adherence class – N (%)				
0%	495 (6.4)	951 (9.1)	1359 (18.9)	604 (12.9)
1-40%	1011 (13.4)	1088 (14.1)	481 (14.1)	447 (17.9)
41-80%	721 (16.5)	816 (22.3)	521 (23.8)	140 (12.8)
>80%	4031 (23.2)	2912 (24.8)	1229 (19.8)	651 (23.3)
Co-medication days per adherence class – N (% [b])				
1-40%	452 (34.4)	389 (30.8)	348 (57.3)	252 (61.2)
41-80%	750 (27.7)	982 (42.7)	777 (59.6)	285 (43.7)
>80%	7697 (45.4)	6041 (52.9)	3148 (52.1)	1844 (67.6)

[a] Group corresponding to patients using any kind of rhinitis medication and, therefore, not corresponding to the sum of weeks and users using oral antihistamines, intranasal corticosteroids and azelastine-fluticasone; [b] Percentage of the days in which each medication class is used.
